# A CoD-based stationary control policy for intervening in large gene regulatory networks

**DOI:** 10.1186/1471-2105-12-S10-S10

**Published:** 2011-10-18

**Authors:** Noushin Ghaffari, Ivan Ivanov, Xiaoning Qian, Edward R Dougherty

**Affiliations:** 1 Department of Electrical and Computer Engineering, Texas A&M University, College Station, TX, 77843 USA; 2 Department of Veterinary Physiology and Pharmacology, Texas A&M University, College Station, TX 77843, USA; 3 Department of Computer Science and Engineering, University of South Florida, 4202 E Fowler Ave., ENB 118, Tampa, FL 33620, USA; 4 Translational Genomics Research Institute (TGEN), 400 North Fifth Street, Suite 1600, Phoenix, AZ 85004 USA

## Abstract

**Background:**

One of the most important goals of the mathematical modeling of gene regulatory networks is to alter their behavior toward desirable phenotypes. Therapeutic techniques are derived for intervention in terms of stationary control policies. In large networks, it becomes computationally burdensome to derive an optimal control policy. To overcome this problem, greedy intervention approaches based on the concept of the Mean First Passage Time or the steady-state probability mass of the network states were previously proposed. Another possible approach is to use reduction mappings to compress the network and develop control policies on its reduced version. However, such mappings lead to loss of information and require an induction step when designing the control policy for the original network.

**Results:**

In this paper, we propose a novel solution, CoD-CP, for designing intervention policies for large Boolean networks. The new method utilizes the Coefficient of Determination (CoD) and the Steady-State Distribution (SSD) of the model. The main advantage of CoD-CP in comparison with the previously proposed methods is that it does not require any compression of the original model, and thus can be directly designed on large networks. The simulation studies on small synthetic networks shows that CoD-CP performs comparable to previously proposed greedy policies that were induced from the compressed versions of the networks. Furthermore, on a large 17-gene gastrointestinal cancer network, CoD-CP outperforms other two available greedy techniques, which is precisely the kind of case for which CoD-CP has been developed. Finally, our experiments show that CoD-CP is robust with respect to the attractor structure of the model.

**Conclusions:**

The newly proposed CoD-CP provides an attractive alternative for intervening large networks where other available greedy methods require size reduction on the network and an extra induction step before designing a control policy.

## Introduction

A key purpose of modeling gene regulation via gene regulatory networks (GRNs) is to derive strategies to shift long-run cell behavior towards desirable phenotypes. To date, the majority of the research regarding intervention in GRNs has been carried out in the context of probabilistic Boolean networks (PBNs) [1]. Assuming random gene perturbation in a PBN, the associated Markov chain is ergodic, and thus it possesses a steady-state distribution (SSD), and (from a theoretical standpoint) one can always change the long-run behavior using an optimal control policy derived via dynamic programming [2,3]. In practice, however, the computational requirements of dynamic programming limit this approach to small networks [4,5]. As an alternative to such optimal intervention, greedy control approaches using mean-first-passage time (*MFPT-CP* algorithm) or the steady-state distribution directly (*SSD-CP* algorithm) have been proposed (CP denoting control policy) [6,7]; nonetheless, these algorithms have their own computational issues owing to their need to use the state transition matrix (STM) of the Markov chain. To overcome the computational problems associated with the design of control policies for larger PBNs, previous studies have proposed reduction mappings that either delete genes [8] or states [9]. Deletion of network components compresses large networks, but at the cost of information loss. Furthermore, reduction mappings themselves can be computationally demanding [8,9].

The control approach taken in this paper circumvents many of the computational impediments of previous methods by basing its intervention strategy directly on inter-predictability among genes. Referring to a gene that characterizes a particular phenotype as a *Target* (*T*) gene and a gene used to alter the long-run behavior of the network by controlling the expression of *T* as a *Control* (*C*) gene, the method proposed herein relies on the predictive power of a small group of genes, which includes the control gene, and designs a stationary control policy that alters the steady-state distribution of the model. The algorithm is designed for the specific class of networks where there is a path from the control to the target gene – an assumption having a natural interpretation in terms of the biochemical regulatory pathways present in cells. Our method simplifies the procedure of designing the stationary control policy and eliminates the need to have a complete knowledge about the STM. Most importantly, the new algorithm can be used to design stationary control policy directly on large networks without deleting any genes/states. It only requires knowledge about the SSD of the network which can be estimated without inferring the STM. The coefficient of determination (CoD) is used for measuring the power of gene interactions [10]. Thus, our new algorithm is optimized for and performs especially well on network models that are inferred from data using CoD-based approaches, e.g. the well-known seed-growing algorithm [11]. The proposed algorithm, called *CoD-CP* because the CoD is the main tool, uses the marginal probabilities of the individual genes obtained from the steady-state distribution of the network to calculate the CoDs.

The most important advantage of the proposed *CoD-CP* is that it can be designed on networks with many genes, and without any compression of the model. All of the previously proposed methods for working with large GRNs, e.g. *CoD-Reduce*[*8*] or state reduction [9], require ‘deletion’ of network components to achieve a compressed model, which allows for the design of the control policy. An induction step is then required in order to induce those control policies back to the original networks. In this paper, we propose a new approach, which designs control policies directly on the original network and requires neither reduction/compression nor induction.

We performed a series of simulation studies to validate *CoD-CP* performance. Our experiments show that in small networks, where it is possible to derive the currently available greedy MFPT-CP [6] and SSD-CP [7] policies, *CoD-CP* achieves a similar performance. Most importantly, when the size of the network is large and MFPT-CP or SSD-CP cannot be designed directly on the original model, *CoD-CP* is easily constructed and applied to the network without any reduction mappings and induction of the control policy from the reduced network back to the original model. Section describes our simulations results. When the network is large, a reduction step is needed before designing the MFPT-CP or SSD-CP. In these cases, *CoD-CP* can be designed directly on the large networks and performs better than the induced MFPT-CP and SSD-CP on average for networks with singleton attractors only or models where cyclic attractors are allowed. We examined *CoD-CP* performance for two different perturbation probabilities and the results show consistent patterns. Furthermore, we examined the performance of the three algorithms on a 17-gene gastrointestinal cancer network derived from microarray data. *CoD-CP* designed on that model network outperforms the stationary MFPT-CP and SSD-CP policies induced from the reduced versions of the 17-gene model. Thus, our new approach provides an attractive alternative to the methods that require network reduction and an extra induction step before designing a control policy.

## Background

### Boolean networks

A *Boolean network with perturbation p*, *BN_p_* = (*V*, **f**), on *n* genes is defined by a set of nodes *V* = {*x*_1_, …, *x_n_*} and a vector of Boolean functions **f** = [*f*^1^, …, *f^n^*]. The variable *x_i_* ∈ {0,1} represents the expression level of gene *i*, with 1 representing high and 0 representing low expression [12]. **f** represents the regulatory rules between genes. At every time step, the value of *x_i_* is predicted by the values of a set, *W_i_*, of genes at the previous time step, based on the regulatory function *f^i^*. *W_i_* = {*x*_*i*_1__, …, *x*_*i*_*k*_*i*___.} is called the *predictor set* and the function *f^i^* is called the *predictor function* of *x_i_*. A state of the *BN_p_* is a vector **s** = (*x*_1_, …, *x_n_*) ∈ {0, 1}*^n^*, and the *state space* of the *BN_p_* is the collection *S* of all possible network states. The perturbation probability *p* ∈ (0,1] models random gene mutations, i.e. at each time point there is a probability *p* of any gene changing its value uniformly randomly. The underlying model of a *BN_p_* is a finite Markov chain and its dynamics are completely described by its 2*^n^* × 2*^n^* state transition matrix, , where *p*(**s***_i_*, **s***_j_*) is the probability of the chain undergoing the transition from the state **s***_i_* to the state **s***_j_.* The perturbation probability *p* makes the chain ergodic and therefore it possesses a steady-state probability distribution *π* which satisfies [13]:(1)

### Coefficient of determination (CoD)

The *coefficient of determination* (CoD) measures how a set of random variables improves the prediction of a target variable, relative to the best prediction in the absence of any conditioning observation [10]. Let **X** = (*X*_1_, *X*_2_, …, *X_n_*) be a vector of binary predictor variables, *Y* a binary target variable, and *f* a Boolean function such that *f*(**X**) predicts *Y.* The mean-squared error (MSE) of *f*(**X**) as a predictor of *Y* is the expected squared difference, *E*[|*f*(**X**) – *Y*|^2^]*.* Let *ε_opt_*(*Y*, **X**) be the minimum MSE among all predictor functions *f*(**X**) for *Y* and *ε*_0_(*Y*) be the error of the best estimate of *Y* without any predictors. The CoD is defined as(2)

Letting **x**_1_, **x**_2_, …, **x**_2^*n*^_ denote the 2*^n^* possible values for **X**, running from (0, 0, …, 0) to (1, 1, …, 1), the relevant quantities are given by(3)

and(4)

[10]. The CoD can be used to measure the strength of the connection between a target gene and its predictors and has been used since the early days of DNA microarray analysis to characterize the nonlinear multivariate interactions between genes [14]. More recently, CoD was used to characterize canalizing genes [15] and contextual genomic regulation [16]. We have restricted ourselves to the Boolean case, thereby arriving at the preceding representations of *ε_opt_*(*Y*,*X*) and *ε*_0_(*Y*)*;* however, the basic definition for *CoD_X_*(*Y*) is not so restricted [10].

### MFPT control policy (MFPT-CP)

Optimal intervention is usually formulated as an optimal stochastic control problem [4]. We focus on intervention via a single control gene *c*, and stationary control policies *µ_c_* : *S* → {0,1} based on *c*. The values 0/1 are interpreted as off/on for the application of the control: 1 meaning that the current value of *c* is flipped, and 0 meaning that no control is applied.

The *mean-first-passage-time* (*MFPT*) policy is based on the comparison between the MFPT of a state **s** and its flipped (with respect to *c*) state [6]. When considering intervention the state space *S* can be partitioned into desirable (*D*) and undesirable (*U*) states according to the expression values of a given *target* set *T* of genes. For simplicity, we assume *T* = {*t*}, the target gene *t* is the leftmost gene in the state’s binary representations, i.e. *x*_1_ = *t*, **s** = (*t*, *x*_2_, …, *x_n_*), and the desirable states correspond to the value *t* = 0. With these assumptions, the state transition matrix *P* of the network can be written as(5)

Using this representation, one can compute the mean-first-passage-time required for a state **s** to reach the boundary between desirable and undesirable states. Computation of these average times is performed in the time scale used for the state transitions of the network. If one uses the states of the network to index the components of the vectors in the 2*^n^* dimensional Euclidean space ℝ^2^*^n^*, then one can form the vectors *K_U_* and *K_D_* that contain the mean-first-passage-time needed for the states in *D* and *U* to reach the undesirable and the desirable states, respectively. For example, the co-ordinate *K_D_*(**s**) of *K_D_* gives the mean-first-passage-time for the undesirable state **s** to reach the set *D* of desirable states. The two vectors *K_U_* and *K_D_* are of dimension 2*^n^*^ – 1^, and, according to a well-known result from the theory of Markov chains [13], are given as solutions to the following system of linear equations:(6)(7)

where *e* denotes the vector of dimension 2*^n^*^ – 1^ with all of its co-ordinates equal to 1.

To understand the intuition behind the MFPT-CP algorithm it is important to notice that, because the control gene *c* is different from the target gene, every state **s** belongs to the same class of states, *D* or *U*, as its flipped state . With this in mind, if a desirable state **s** reaches *U* on average faster than , it is reasonable to apply control and start the next network transition from its flipped state . Thus, the design of the stationary MFPT-CP is based on the differences  and . The MFPT-CP algorithm uses a tuning parameter *γ* > 0, and these differences are compared to the value of *γ*, which is related to the cost of applying control. For example, *γ* is set to a larger value when the ratio of the cost of control to the cost of the undesirable states is higher, the intent being to apply the control less frequently [6].

The MFPT concept could be used in two different ways to design the intervention strategy. The first approach is called “model-dependent” and needs the state transition matrix of the Markov Chain. The time-course measurements can be used to estimate the transition probabilities for all states. Then the STM is used to find the *K_U_* and *K_D_* vectors to design the control policy. In the second approach, called “model-free,” the MFPTs are directly estimated from the time-course data and the inference of the STM is skipped. In this paper we focus on the model-dependent MFPT-CP.

### SSD control policy (SSD-CP)

The *steady-state-distribution* (*SSD-CP*) policy [7] uses the steady-state distribution of a perturbed Markov chain given in [17] to quantify the shift in the steady-state mass after applying possible controls. A perturbation (change) in the logic defining the Boolean network changes the original transition probability matrix *P* and steady-state distribution *π* to  and , respectively. In [17], the fundamental matrix, *Z*, is used to represent  in terms of *π*. *Z* = [*I* – *P* + *eπ^T^*]*^–^*^1^, where *T* denotes transpose and *e* is a column vector whose components are all unity [18]. For a *rank-one perturbation*, the perturbed Markov chain has the transition matrix , where *a*, *b* are two arbitrary vectors satisfying *b^T^e* = 0, and *ab^T^* represents a rank-one perturbation to the original Markov chain *P.* In the special case where the transition mechanisms before and after perturbation differ only in one state, say state **k**,(8)

where *β^T^* = *b^T^Z* and *e*(**k**) is the elementary vector with a 1 in the **k**th position and 0s elsewhere [17-19]. To define the SSD-CP policy let  be the flipped state (with respect to control gene *c*) corresponding to state **s** (as with MFPT-CP). Let *π_U_* be the original steady-state mass of the undesirable states and let  and  denote the steady-state masses of the undesirable states resulting from altering the original state transition matrix by changing the starting state for the next transition from **s** to  and from  to **s**, respectively. The SSD-CP policy is defined on pairs of states, **s** and , in the following manner: if both  and  are larger than *π_U_*, then control is applied to neither; otherwise, if , then control is applied to **s**, and if , then control is applied to .

### Two step design of control policy: reduction followed by induction

The derivation of the optimal or greedy control policies becomes infeasible as the number of genes in the GRN increases. As a solution, deleting the genes is proposed by methods outlined in [8]. The idea is to delete genes sequentially until the size of the network is small enough for designing the control policy. Because the dimension of the control policy designed on the reduced network is not compatible with the original network, it is necessary to induce the control policy from the reduced network to the original one. The best candidate gene for deletion is selected by an algorithm that measures strength of gene-connectivity using the CoD. Genes not predicting any other genes or being predicted by any other genes are called *constant genes* and are the first choice for deletion. If there are not any *constant genes*, then the gene that has minimum CoD for predicting the target gene is selected as the best candidate, *d*, for deletion. After selecting *d*, a reduction mapping is used to define the transition rules for states in the reduced network [20]. The design of the reduction mapping is based on the notion of a *selection policy*[*8*]. A *selection policy ν^d^* corresponding to the deleted gene *d* is a 2*^n^* dimensional vector, *ν^d^* ∈ {0, 1}^2^*^n^*, indexed by the states of *S* and having components equal to 1 at exactly one of the positions corresponding to each pair **, s** ∈ *S***.** For each gene *d* there are 2^2 – *n*^ different selection policies.

Since finding the optimal selection policy is computationally impossible in large GRNs, an heuristic approach is proposed by [8]: if either state **s** or  is an attractor, then the attractor state is chosen to determine the function structure, but if neither is an attractor, then the transitions of the state possessing larger steady-state probability mass are kept as transitions for the reduced state.

Finally, after a control policy designed on the reduced network, it is necessary to induce it back to the original model. The induction procedure repeats the same control action for the two states  and **s** that collapsed together to form the **š** states in the reduced network. The induction is formally defined as follows, where *n* is the number of genes in the original model. Assume that after *n* – *m* gene deletions the reduced network has *m* <*n* genes. Then, for any state (*x*_1_, *x*_2_, …, *x_m_*) in the reduced network, there are 2*^n^*^–^*^m^* states in the original network of the form (*x*_1_, …, *x_m_*, *z*_1_, …, *z_n_*_–_*_m_*). If *µ_red_* is the control policy designed on the reduced network, then the induced policy on the original network is defined by(9)

for any *z*_1_, …, *z_n–m_* ∈ {0,1}.

## Proposed methodology

This section describes our new algorithm, *CoD-CP.* The algorithm takes advantage of the predictive power of triplets of genes that include the control gene to predict the expression of the target gene with a small estimated error. To achieve the best performance of the algorithm, it is necessary to have a direct connection or a path from the control gene to the target gene in the regulatory network. The algorithm uses the CoD to measure that predictive power and to design a control policy.

*CoD-CP* is a greedy technique for designing a stationary control policy. The target gene defines the phenotype and divides states into two mutually disjoint sets, *D* (desirable) and *U* (undesirable). The gene with the most predictive power over the target gene *T* among the genes connected with a path to *T* is used as the control gene *C.* The goal of the algorithm is to increase the total probability mass of desirable states in the long-run by controlling *C.*

*CoD-CP* starts by generating all 3-gene combinations that include *C.* We use three genes for predicting *T* because, as Kauffman points out, the average connectivity of the model cannot be too high if its dynamics are not chaotic [21] and 3-gene predictors are commonly assumed in BN and PBN modeling [1]. *CoD-CP* uses the CoDs for determining the strength of the connection between a target gene and its predictors. The CoDs are calculated using the SSD of the network and the respective conditional probability distribution (CPD) tables. After examining all 3-gene combinations, they are sorted based on their CoDs. The triple that has the maximum CoD with respect to *T* and its corresponding CPD is stored and used for designing the control policy. If there is more than one such a triple, we can uniformly randomly decide to use one of them. We refer to this triple as *MAXCOD* and its CPD is called *MAXCPD*. Table 1 represents an example of a *MAXCPD* table, where the first three columns contain the binary combinations of the *MAXCOD* genes. Using *T* and the *MAXCOD* genes, the state space of the network is broken down into blocks with 2*^n^*^ – 4^ states. All states in a *block* share the same values for *T* and the *MAXCOD* genes. The details about the entries of the *MAXCPD* table are given in the Example 1, part a.

**Table 1:** MAXCPD Table: the first three columns represent the binary combinations of the three MAXCOD genes. The last two columns are filled by summing up the SSD probabilities of states in each corresponding block.

	*MAXCOD*			*T*	
	
	*C*	*Predictor 1*	*Predictor 2*	0	1
	
row 1	0	0	0	*P*_10_	*P*_11_
	
row 2	0	0	1	*P*_20_	*P*_21_
	
row 3	0	1	0	*P*_30_	*P*_31_
	
row 4	0	1	1	*P*_40_	*P*_41_
	
row 5	1	0	0	*P*_50_	*P*_51_
	
row 6	1	0	1	*P*_60_	*P*_61_
	
row 7	1	1	0	*P*_70_	*P*_71_
	
row 8	1	1	1	*P*_80_	*P*_81_

***Example 1*, *part a*:** This example explains the entries of the *MAXCPD* table using a 7-gene network with 128 states. Without loss of generality, assume that *x*_1_ and *x*_2_ are the *T* and *C* genes, respectively, and *x*_1_ = 0 defines desirable states. After examining all the triples, *MAXCOD* is found to be {*x*_2_, *x*_3_, *x*_4_}, which has maximum CoD for predicting *x*_1_. The first three columns of the *MAXCPD* table contain 8 binary combinations of *x*_2_, *x*_3_ and *x*_4_, as table 1 shows. The last two columns of the table contain the summation of the SSD probabilities of the states with common value for *MAXCOD* genes. The only difference in columns four and five is the value of the *T* gene. The size of each block of states is 2*^n^*^– 4^ = 2^3^ = 8. The first block is *Block*(1) = {0000000, 0000001, 0000010, 0000011, 0000100, 0000101, 0000110, 0000111}, where all have {*x*_2_, *x*_3_, *x*_4_} = 000 and *x*_1_ = 0. The second block is *Block*(2) = {1000000, 1000001, 1000010, 1000011, 1000100, 1000101, 1000110, 1000111}, where {*x*_2_, *x*_3_, *x*_4_} = 000 and *x*_1_ = 1. Each entry of the forth and fifth columns of the CPD table are represented by *P_ij_*, where *i* ∈ {1, …, 8} represents a row and *j* ∈ {0,1} is the *T* value. Each *P_ij_* is the summation of the SSD probabilities of the states in a block. For columns four and five of the first row (*i* = 1), we have to sum up all the SSD probabilities for the states in *Block(1)* to find *P*_10_*.* The summation of the SSD probabilities of *Block(2)* forms *P*_11_*.* The rest of the *P_ij_*s are calculated similarly.

In the PBN setting, control of the network is achieved by toggling the value of the control gene. The derivation of a stationary control policy *µ* ∈ {0, 1}^2^*n*^^, means defining control actions for each state **s** ∈ {*StateSpace*}*.* If the control action for the state **s** is set to 1, it means that the network should transition from its flipped with respect to . Otherwise the network transitions as specified by its STM. The *CoD-CP* algorithm finds the *MAXCPD* table in order to specify the control actions. It uses the total probabilities *P_ij_* to define the control actions. Algorithm 1 details all the steps of *CoD-CP.* In the binary representation of each state **s**, we find the values of *MAXCOD* genes. The decimal conversion of the values of *MAXCOD* genes determines the row of the *MAXCPD* table corresponding to state **s**. Then, the total probabilities *P_ij_* are used to find *D*(*.*), as described by algorithm 1, where *D*(*.*) defines the difference between the total probability of a block of states to be desirable from that of being undesirable in the long run. Using this difference we can define the control actions: if , then flip the value of *C* in  to start the transition from **s**; otherwise, flip the value of *C* in **s** and start the next transition of the Markov chain from . If , then we can select one of them uniformly randomly. Example 1, part b illustrates how control actions are assigned to the states.

**Figure 1:**
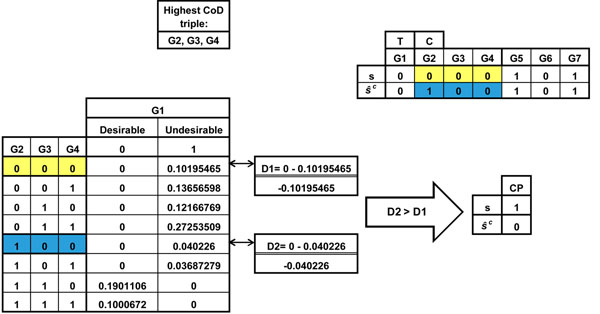
Deriving CoD-CP for a small 7-gene network. The *x*_1_ and *x*_2_ genes are the *T* and *C* genes, respectively. *x*_1_ = 0 defines *Desirable* states. The *MAXCOD* genes are: {*x*_2_, *x*_3_, *x*_4_}. The control action for state s is 1 and the control action for state  is 0, because *D*(*2*) >*D*(*1*).

***Example 1*, *part b:*** Following the same 7-gene example, consider state **s** = 0000000. We calculate *D*(**s**) = *P*_10_ – *P*_11_*.* The flipped state with respect to the control gene is . Looking at the *MAXCOD* genes in the binary representation of , we have {*C* = 1, *Predictor*1 = 0, *Predictor*2 = 0}, which maps to row 5 of the *MAXCPD* table. Similarly, . If , then it is beneficial to flip  and force the Markov chain to start the next transition from **s**, but if , then it is better to start the next transition from , in which case the control action for **s** is set to 1. For all the states in *Block*(*1*) the same control action is applied. This greatly simplifies the design of the control policy. Figure 1 shows a numerical example of how the *CoD-CP* can be designed on this 7-gene example network.

## Performance comparison

In this section we compare the performances of *CoD-CP*, SSD-CP, and MFPT-CP, first with respect to run time and then to shift of the steady-state distribution.

### Run-time comparison

The dynamics of a GRN and its associated Markov chain are determined by its state transition matrix. The STM provides the full knowledge about the states and their transitions in the network; however, inferring the STM is difficult, especially when available data about the network are limited or the size of the network is large. The main advantage of the *CoD-CP* algorithm is that it can be directly designed on large networks without inferring the STM and only needs an estimation of the SSD of the Markov chain. This section provides a comparison of *CoD-CP* with MFPT-CP [6] and SSD-CP [7].

**Figure 2:**
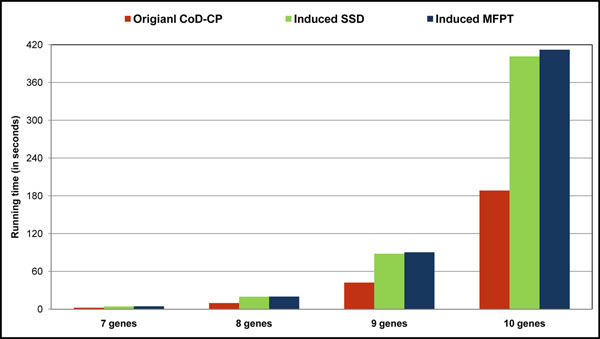
Comparing the average running times(in seconds) for designing stationary control policy for 100 randomly generated 10-gene, 9-gene, 8-gene and 7-gene *BN_p_s*. Running time for CoD-CP algorithm is always less than MFPT-CP and SSD-CP. The running time grows exponentially as the number of genes increases.

In the case of large GRNs, *CoD-CP* can be directly designed on the model, while MFPT-CP and SSD-CP are two-step procedures: first reducing the size of the network so that the policy can be designed and then inducing that control policy back to the original network. These necessary steps increase the computational time associated with MFPT-CP and SSD-CP. To compare the three algorithms, we measured the running time needed for designing control policies on gene networks containing 7, 8, 9, and 10 genes, averaged for 100 randomly designed *BN_p_s*. For MFPT-CP and SSD-CP, the best gene for deletion was selected and then the original network was reduced by deleting that gene, according to the methodology introduced in [8]. Consequently, the control policies were designed on the reduced networks and then induced back to the original networks. *CoD-CP* was designed directly on the original network as described by our new algorithm. All computations were performed on a computer with 4GB of RAM and Intel(R) Core(TM) i5 CPU, 2.53 GHz. Figure 2 shows the average running times for 100 *BN_p_s* in seconds. The running times tend to grow exponentially as the number of genes increases.

For comparing the performance of the three algorithms one needs to keep in mind their important characteristics. The *CoD-CP* algorithm needs the SSD to design the control policy. In cases when the SSD is known, one can directly proceed to the CoD calculations and design the control policy for the network. When the SSD is not known, it can be calculated using equation (1) or can be estimated by methods described in [22]. The model-dependent version of the MFPT algorithm requires an extra step to infer the STM. It then uses matrix inversion to find the mean-first-passage-time vectors *K_D_* and *K_U_*, this step having the same time complexity as finding the SSD. The model-free version of MFPT-CP requires time-course measurements to estimate the necessary mean-first-passage time vectors. In such a case the algorithm can skip the inference of the STM, and the complexity of estimating MFPT vectors is constant with respect to the number of genes. However, the availability of time-course data is very limited in practice. The other available greedy approach, SSD-CP also requires the SSD and STM of the network. Moreover, the SSD-CP algorithm needs to find the perturbed SSD for each state, which increases the time spent for designing the control policy.

As described in the section , *CoD-CP* uses the *MAXCPD* table to design the control policy, which divides the state space into blocks of size 2*^n^*^– 4^. These blocks are used to assign the same control actions to all of the states in a given block and the complement control action for the block of flipped states. This significantly reduces the complexity of the control policy design and leads to shorter run times.

### Steady-state performance

This section provides simulation experiments to demonstrate the performance of the *CoD-CP* algorithm with respect to its main goal, to shift undesirable steady-state mass to desirable steady-state mass. In the first part, the algorithm is applied to randomly generated networks. In the second part, we demonstrate *CoD-CP* on a real-world-derived gastrointestinal cancer network with 17 genes, which can be considered large, given that even with binary quantization, the dimension of its Boolean network STM is 2^17^ × 2^17^.

### Synthetic networks

*CoD-CP* has been designed for networks that are too large for direct application of greedy algorithms such as MFPT-CP and SSD-CP while at the same time not suffering from loss of information when designing control polices on reduced networks and then inducing them to the corresponding original networks. Hence, our desire is to demonstrate the improved performance of *CoD-CP* in comparison to the induced greedy control policies when reduction-inducement is necessary; otherwise, one can simply use the previously developed greedy policies directly. In this section, we discuss the results of a simulation study that compares the performance of *CoD-CP* to MFPT-CP and SSD-CP on a set of *BN_p_s* that are randomly generated using the algorithm from [23], for two different perturbation probabilities: *p* = 0*.*1 and *p* = 0*.*01. The latter probability is the one most commonly used in GRN control studies [2, 6, 7, 24]; nevertheless, we also use *p* = 0*.*1 to see the effect, if any, of a less stable network where less mass is concentrated in the attractors. In order to examine how the attractor structure affects performance, we test the *CoD-CP* algorithm on two model classes: (1) networks with singleton attractors only, and (2) networks that allow cyclic attractors. In the first class, we randomly choose 100 unique attractor sets for a different number of genes *n*, where *n* ∈ {7, 8, 9,10}. The attractor sets are restricted to be evenly distributed between the desirable and undesirable states. In the second class, the attractor sets are unique, but the criteria for evenly distribution between *D* and *U* is no longer required and attractors are allowed to be cyclic and of unequal length. We used the *absolute shift* of the SSD as the algorithm performance measure. It is given by(10)

Where  and  are the total probability masses of the desirable states after applying control and before applying any control, respectively, a larger *λ* being desirable. In real-world situations the target (*T*) and control (*C*) genes are often pre-selected by the biologists/clinicians, the basis for choice being that a phenotypically related target is to be up- or down-regulated and the control gene is known to be related to the target. However, in our simulation studies, where knowledge about *T* and *C* does not exist, we have designed a procedure to identify reasonable target and control genes. The objective of the procedure is to select a (*C*, *T*) pair such that there is a direct connection, or path, from *C* to *T*, which would be a natural constraint in applications. The strength of connection between *C* and *T* is measured by the CoD. The selected pair is called *CoD-strongly-connected* pair. To select this pair, we consider all two-gene combinations such that each gene in a given pair is treated as both the candidate target and candidate control gene, and the CoD of the candidate *C* for predicting candidate *T* is calculated. The pair with the maximum CoD of *C* candidate for predicting candidate *T* is picked. Then the algorithm checks if there is a path from *C* to the *T*. If such a path exists, then the (*C*, *T*) pair is chosen. If no path exists, then the pair is discarded and the next highest CoD pair is considered as the candidate (*C*,*T*) pair. For checking the existence of a path, we use the breadth-first-search (BFS) algorithm [25]. For more information please refer to the supplemental document (Additional file 1).

**Figure 3:**
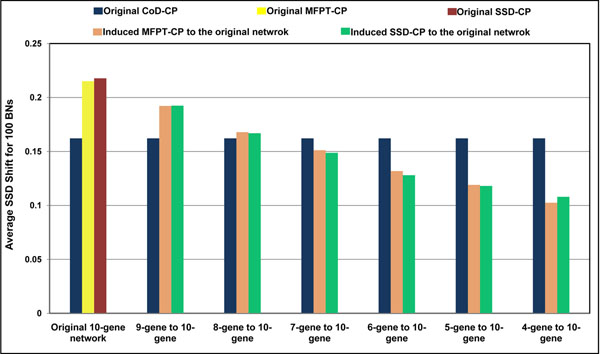
Comparing original CoD-CP to the original and induced MFPT-CP and SSD-CP for 100 randomly generated 10-gene *BN_p_s* with half of the attractors in *D* states. In the first set of bars, CoD-CP, MFPT-CP and SSD-CP are designed on the 10-gene networks. In the next sets, the CoD-CP was designed on the original 10-gene networks and compared to the induced MFPT-CP and SSD-CP. At each step, one gene was deleted, and then MFPT-CP and SSD-CP were designed and induced back to the original network, until each *BN_p_* had only 4 genes. The perturbation probability is 0.1.

**Figure 4:**
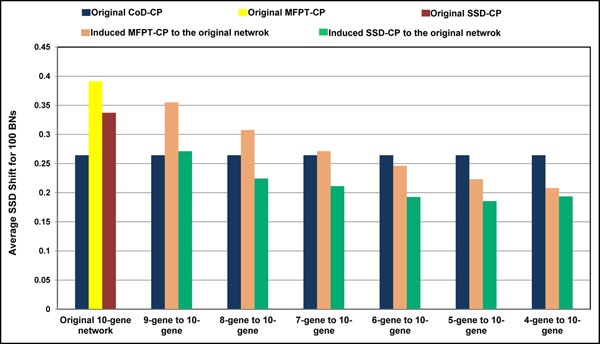
Comparing original CoD-CP to the original induced MFPT-CP and SSD-CP for 100 randomly generated 10-gene *BN_p_s* with half of the attractors in *D* states. In the first set of bars, CoD-CP, MFPT-CP and SSD-CP are designed on the 10-gene networks. In the next sets, the CoD-CP was designed on the original 10-gene networks and compared to the induced MFPT-CP and SSD-CP. At each step, one gene was deleted, and then MFPT-CP and SSD-CP were designed and induced back to the original network, until each *BN_p_* had only 4 genes. The perturbation probability is 0.01.

To compare *CoD-CP* to the reduction-inducement versions of MFPT-CP and SSD-CP, we use the reduction method described in [8], called *CoD-Reduce*. The *CoD-Reduce* algorithm is designed for the networks with singleton attractors only because its selection policy heuristically uses the singleton attractors to generate the structure of the reduced network. Therefore, in this paper, when reduction of the network is needed for comparison of the control policies, we focus on the networks with singleton attractors only (we return to cyclical attractors later). Figure 3 illustrates that the *CoD-CP* policy designed on the original network outperforms the induced MFPT-CP and SSD-CP policies when there is significant network reduction in the case of a 10-gene network and *p* = 0*.*1. Each set of bars in the graph shows the average SSD shifts for the three policies with different amounts of reduction for the MFPT-CP and SSD-CP policies, beginning no reduction-induction, then reduction to 9 genes and induction back to 10, and so on. The performance of *CoD-CP* is invariant because it is designed directly from the original network. Absent reduction we see that *CoD-CP* is outperformed by the induced polices and continues to be outperformed with a 2-gene reduction. But after that, for reductions of 3 ore more genes, *CoD-CP* outperforms the induced policies, with its superiority increasing as the extent of the reduction grows. This is precisely the behavior we desire. While both the MFPT-CP and SSD-CP policies can be used directly for 10-gene networks, they must be induced from reductions for large networks and, as we observe, the reduction-induction paradigm provides decreasing SSD shift as the amount of reduction increases. Figure 4 shows a similar phenomenon with *p* = 0*.*01.

Having demonstrated the advantage of *CoD-CP* over the induced polices as the degree of reduction (and, therefore, induction) increases, we now turn to two other aspects of *CoD-CP*: the effect of cyclic attractors and the selection of target-control pairs. For each issue we consider two cases. For attractors, as previously noted, we have: (a) only singleton attractors and (b) cyclic attractors allowed. Regarding target-control pairs, we have: (a) *CoD-strongly-connected* target-control pairs and (b) randomly selected target-control pairs. If we combine these choices, we have four factors to consider: network size (*n*), perturbation probability (p), attractor structure, and target-control structure. Table 2 provides the SSD shifts for network size *n* ∈ {7, 8, 9,10}, *p* ∈ {0.1, 0.01}, and the two possibilities for attractors and target-control pairs.

**Table 2:** CoD-CP performance for *p* ∈ {0.1, 0.01} and singleton or cyclic attractors, averaged for 100 *BN_p_s* with 7, 8, 9 and 10 gene networks.

			Network Size			
p	C-T Pair	Attractors	7	8	9	10

0.01	Connected	Singleton	0.442813327	0.43722164	0.343500124	0.26431826

0.01	Connected	Cyclic	0.438436274	0.309464685	0.288786759	0.204716543

0.01	Random	Singleton	0.117863711	0.148585675	0.160006514	0.102484183

0.01	Random	Cyclic	0.069442199	0.068265255	0.116798596	0.06892594

0.1	Connected	Singleton	0.322014658	0.256250706	0.205475527	0.162102211

0.1	Connected	Cyclic	0.315366842	0.237692755	0.191486782	0.132842645

0.1	Random	Singleton	0.072929047	0.069787742	0.069085431	0.043901727

0.1	Random	Cyclic	0.078443655	0.045092185	0.057163771	0.044105186

The first point to recognize is that using *CoD-strongly-connected* target-control pairs is more realistic because in practice one would control a target with gene that is strongly connected to it via prediction and the CoD is a measure of prediction. On the other hand, one could hardly expect to achieve as good results by randomly selecting targets and controls. We see this contrast reflected in the SSD shifts in Table 2. In addition, we see that using *CoD-strongly-connected* target-control pairs results in decreasing SSD shift for increasing network size, whereas this trend is replaced by sporadic behavior for randomly selected target-control pairs. Finally, we note the better performance for *p* = 0*.*01 than for *p* = 0*.*1. This reflects the more random network behavior for higher perturbation probability because the control algorithm utilizes the predictive structure in the network (as measured by the CoD) and this structure is less determinative when perturbations are more likely. In this regard we note that both MFPT-CP and SSD-CP also perform better for *p* = 0*.*01 than for *p* = 0*.*1, in both their non-induced and induced modes.

### Gastrointestinal cancer network

**Figure 5:**
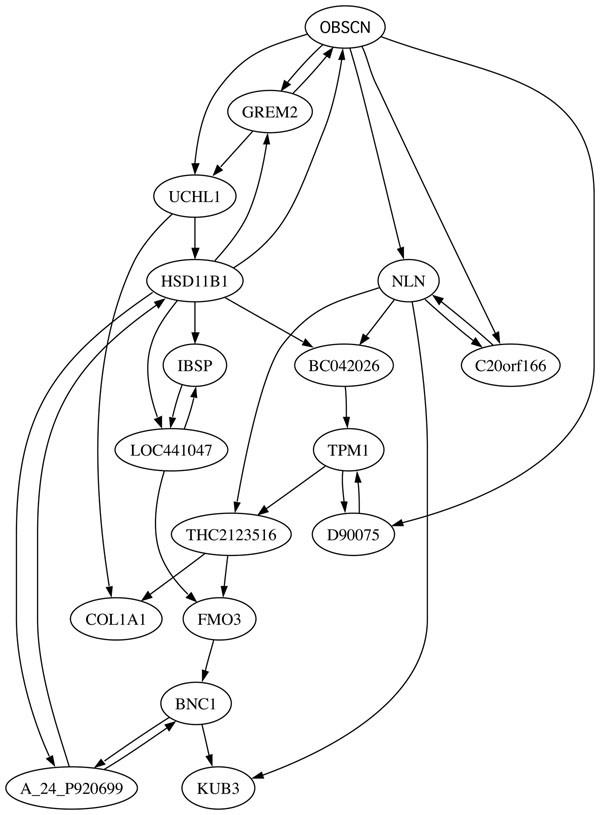
17-gene Gastrointestinal Cancer Network. Details about the network and this graphical display are provided in [8,9]

This section accomplishes two purposes: to examine *CoD-CP* performance on a real data-based network and on a network sufficiently large that neither MFPT-CP nor SSD-CP (nor, for that matter, dynamic programming) can be applied in their non-induced forms. To do so, we use a *BN_p_* derived from gastrointestinal cancer microarray dataset [26] and initially inferred in [8]. The 17-gene network has the genes *OBSCN* and *GREM2* as target and control, respectively. This selection is based on biological knowledge and CoD-measured strength of the connectivity between them. The 17 genes comprising the model are: *OBSCN*, *GREM*2, *HSD*11*B*1, *UCHL*1, *A*_24_*P*920699, *BNC*1, *FMO*3, *LOC*441047, *THC*2123516, *NLN*, *COL*1*A*1, *IBSP*, *C*20*or f*166, *KUB*3, *TPM*1, *D*90075, and *BC*042026. Figure 5 shows the connectivity graph of the 17-gene gastrointestinal cancer network.

The 17-gene network has a 2^17^ × 2^17^ state transition matrix. The generation and manipulation of the STM needed for the design of the MFPT-CP and SSD-CP is a hard computational problem, thus, reduction and induction are necessary steps for obtaining the two control policies. We use an estimation of the SSD because for such a large network it is infeasible to derive it analytically. The approximation method proposed in [22] is used to estimate the SSD of the network. Since *CoD-CP* can use the estimated SSD of the network, it can be used for directly designing the stationary control policy on the 17-gene network. The estimation procedure uses the Kolmogorov-Smirnov test to decide if the network has reached its steady-state.

To apply MFPT-CP and SSD-CP, we reduce the network via the gene reduction method introduced in [8] and delete genes consecutively until only 10 genes are left in the network. At that point it is possible to design the MFPT-CP and SSD-CP policies, after which they are induced back to the original 17-gene network. The resulting performance comparison of the *CoD-CP* policy with the induced MFPT-CP and SSD-CP policies is shown in Table 3. The difference is dramatic, with the SSD shift for the *CoD-CP* far superior to the shift for the induced MFPT-CP and SSD-CP policies, which are about the same. The perturbation probability used in this experiment is *p* = 0*.*1. More results using *p* = 0*.*01 are shown by table 4. It is important to point out that the small perturbation probability, *p* = 0*.*01, makes such a large network to be very deterministic. Thus, all of the three control policies produce significant shifts in the network SSD towards the desirable states. In addition, one can notice that the *CoD-CP* performs extremely well which can be attributed to the use of CoD to infer the network structure from data. This results illustrates the importance of the proper combination of network inference and control policy design methods.

**Table 3:** Comparing SSD shift before and after applying control policies, *p* = 0.1. The *CoD-CP* designed on the 17-gene Gastrointestinal cancer network. For MFPT-CP and SSD-CP, The 17-gene network is reduced to 10 genes, the control policies designed for it and then these policies induced back and applied on the original 17-gene network.

Total Probability mass of *D* states, before control	0.499871
Total Probability mass of *D* states, after original CoD-CP	0.726651

Total Probability mass of *D* states, after induced MFPT-CP	0.547329

Total Probability mass of *D* states, after induced SSD-CP	0.548864

**Table 4:** Comparing SSD shift before and after applying control policies, p=0.01. The CoD-CP designed on the 17-gene Gastrointestinal cancer network. For MFPT-CP and SSD-CP, The 17-gene network is reduced to 10 genes, the control policies designed for it and then these policies induced back and applied on the original 17-gene network.

Total Probability mass of *D* states, before control	0.497549
Total Probability mass of *D* states, after original CoD-CP	0.988466

Total Probability mass of *D* states, after induced MFPT-CP	0.788339

Total Probability mass of *D* states, after induced SSD-CP	0.747307

## Conclusions

In this paper we propose a new algorithm, *CoD-CP*, for designing a greedy stationary control policy that beneficially alters the dynamics of large gene regulatory networks. The proposed algorithm needs minimum knowledge about the structure of the model and only uses the steady-state distribution of the associated Markov chain. This is particularly important for large networks, where it is computationally prohibitive to use the previously proposed optimal or greedy approaches for designing stationary control policies. The *CoD-CP* algorithm uses CoD computations based on the steady-state distribution for measuring the strength of connection between the target gene and its candidate predictor genes. *CoD-CP* is particularly designed for the class of network models where there is a path between the target and control genes, a condition that is reasonable in practical applications. The control action for each state of the network is defined based on the values of the strongest predictor set for the target gene. Simulations demonstrate that *CoD-CP* outperforms the induced versions of the MFPT-CP and SSD-CP algorithms relative to shifting the steady-state distribution of the network toward more desirable states when there is a significant amount of reduction, a requirement for large networks.

## Authors contributions

NG proposed the main idea, developed the algorithm, designed and performed the simulations, and prepared the manuscript. II collaborated on the design of the algorithm and simulations, interpretation of the results and manuscript preparation. XQ provided insights on the interpretation of the algorithm and results and helped on the manuscript. ERD conceived the study, participated in the analysis and interpretation of the results, and helped draft the manuscript. All authors read and approved the final manuscript.

## Competing interests

The authors declare that they have no competing interests.

## Supplementary Material

Additional file 1 This is a file in PDF format and contains additional and supportive material. It provides details about SSD estimation methods, compares results from this paper to the previously published results and outlines the method used for selecting *CoD-strongly-connected T-C* pairs in the simulations. The link to the file is: http://gsp.tamu.edu/Publications/supplementary/ghaffari11a/ghaffari-cod-cp-supplemental-document.pdf Click here for file
