# Supplementary material for: A CoD-based stationary control policy for intervening in large gene regulatory networks
Source: BMC Bioinformatics. 2011 Oct 18;12(Suppl 10):S10. doi: 10.1186/1471-2105-12-S10-S10 (PMC3236832; doi:10.1186/1471-2105-12-S10-S10)
Supplement: Additional file 1 — This is a file in PDF format and contains additional and supportive material. It provides details about SSD estimation methods, compares results from this paper to the previously published results and outlines the method used for selecting CoD-strongly-connected T-C pairs in the simulations. The link to the file is: http://gsp.tamu.edu/Publications/supplementary/ghaffari11a/ghaffari-cod-cp-supplemental-document.pdf [file 1471-2105-12-S10-S10-S1.pdf]

Supplemental Document

This is the supplemental document for the *CoD-CP* algorithm proposed by the paper *"A CoD-based Stationary Control Policy for Intervening in Large Gene Regulatory Networks"* by Noushin Ghaffari, Ivan Ivanov, Xiaoning Qian, and Edward R. Dougherty.

I. SSD Estimation Methods

The current paper uses a 17-gene real-world derived network for comparing the performance of the *CoD-CP* with MFPT-CP and SSD-CP. The *CoD-CP* can be used for directly designing the stationary control policy on the 17-gene network and only need the SSD of the network. To compare the performance of the *CoD-CP* designed on the 17-gene network with the MFPT-CP and SSD-CP, we had to reduce the size of the network before being able to design the latter two intervention policies. We used the gene reduction method introduced in [2] and deleted genes consecutively until 10 genes were left in the network. At that point it was possible to design MFPT-CP and SSD-CP; then those policies were induced back to the original 17-gene network. The steady state distribution of the original 17-gene network is needed for designing the *CoD-CP* and also for reduction of the network. Due to the large size of the network it is computationally impossible to analytically derive the SSD of the network. We estimated the SSD of the network using the method proposed in [3]. The intuition behind the algorithm is to let the network transition for a long time and then use the Kolmogorov-Smirnov test to examine if the network reached its steady state. The method for deciding state transitions of the Markov Chain in the current paper differs slightly from the method which is used in [2]. The 17-gene network used in two papers are the same, but it is noted that the SSD shift toward *Desirable* states using induced MFPT-CP is different. The two different state transition methods led to both different steady-states of the networks and differences in the SSD shift after applying a control policy. In this section we describe these two state transition methods.

The main assumption in [2] is that the decision to use either gene perturbation or the network's transition function for the next network transition is made with a probability of 0.5. Such an interpretation leads to using the network transition function approximately half of the time.

If transition using the functions does not happen, then it uses perturbations for each gene to determine the next state of the network. On the other hand, the current paper uses a two-step process to decide the next network transition. First, an indicator random variable that follows the multinomial distribution generated by the individual gene perturbation probabilities is used to check if the network will transition by a random perturbation in one or more genes. If the indicator variable shows that no gene perturbation occurs, then, the network transition function is used for the next network transition. The second approach that is used in the current paper is more widely accepted. This is most commonly accepted transition method for both the BN_p and the *PBN* models.

It is important to note that regardless of which state transition method is used, the currently proposed *CoD-CP* outperforms the induced MFPT control policy.

II. CoD-CP Algorithm: Selecting strongly-connected *T-C* pair as Target and Control Genes for Simulations

In many real-data scenarios the *target* and *control* genes can be specified by biologists/physicians and used directly in deriving the intervention policy.

However, in our simulation studies where the expert knowledge about *target* and *control* genes does not exist, we used the following approach for choosing them.

All of the possible two gene combinations are considered and each gene is examined as either a candidate *T* or a *C* gene. For each two-gene combination, the *CoD* of the candidate *C* for predicting candidate *T* is calculated. The pair with maximum *CoD* of *C* candidate for predicting candidate *T* is selected. Then, the algorithm checks for existence of either direct or indirect path from *C* to the *T*. If a path exist, then the *T-C* pair is chosen as *CoD-Strongly-Connected T-C pair*. However, if there is not such a path, this pair is discarded and the next high *CoD* pair is considered as the candidate *T-C* pair.

For checking of the existence of a *direct path* or *connection* between candidate *T* and *C* genes, the connectivity table of the network is used. This table is built using the truth table of the BN_p as follows: if toggling the value of a predictor gene affects the value of a target genes, then the corresponding entry of the table has 1, otherwise it has 0. Therefore, if there is a *direct path* between *C* and *T* genes, then the corresponding entry in the connectivity table has value 1. This

implies that if for the pair *T-C*, a path from *C* to *T* exists, it also means that control gene can affect the target gene, based on the truth table.

If we cannot find a connection between the *T-C* pair, there is still a possibility of having a *path* which consist of more than these two genes. For checking the existence of an *indirect path(s)*, we used the Breadth-First-Search(BFS) algorithm [1]. The BFS finds all the nodes that are *reachable* from the given source node.

This procedure is repeated until the *CoD-Strongly-Connected T-C pair* is found.

REFERENCES

[1] Cormen, T. H., Leiserson, C. E., Rivest, R. L., and C. Stein, ”Introduction to Algorithms”, *MIT Press and McGraw-Hill*, 2nd edition, ISBN 0-262-53196-8, 2001.

[2] Ghaffari, N., Ivanov I., Quian X. and Dougherty E.R., ”A CoD-based reduction algorithm preserving the effects of stationary control policies for Boolean networks”, *Bioinformatics*, Vol. 26, 1556-1563, 2010.

[3] Kim, S., Li, H., Dougherty, E.R., Chao, N., Chen, Y., Bittner, M.L., and E.B. Suh, ”Can Markov Chain Models Mimic Biological Regulation”, *Biological Systems*, Vol. 10, No. 4, 447-458, 2002.
